# *Trypanosoma cruzi*-infected triatomines and rodents co-occur in a coastal island of northern Chile

**DOI:** 10.7717/peerj.9967

**Published:** 2020-10-14

**Authors:** Ricardo Campos-Soto, Gabriel Díaz-Campusano, Nicol Quiroga, Catalina Muñoz-San Martín, Ninette Rives-Blanchard, Fernando Torres-Pérez

**Affiliations:** 1 Instituto de Biología, Facultad de Ciencias, Pontificia Universidad Católica de Valparaíso, Valparaíso, Chile; 2 Departamento de Ciencias Ecológicas, Facultad de Ciencias, Universidad de Chile, Santiago, Chile; 3 Núcleo de Investigaciones Aplicadas en Ciencias Veterinarias y Agronómicas, Campus Maipú, Universidad de Las Americas, Santiago, Chile; 4 Departamento de Ciencias Biológicas Animales, Facultad de Ciencias Veterinarias y Pecuarias, Universidad de Chile, Santiago, Chile

**Keywords:** *T. cruzi* reservoir in islands, Island *T. cruzi* cycle, Island *T. cruzi* hosts, *Mepraia*, Insular small mammals, Hemiptera:Reduviidae, *Trypanosoma cruzi*, *T. cruzi* life cycle

## Abstract

*Trypanosoma cruzi*, the cause agent of Chagas disease, is transmitted mainly by blood-feeding insects of the subfamily Triatominae. The *T. cruzi* life cycle alternates between triatomines and mammalian hosts, excluding birds and reptiles. Triatomines of *Mepraia* genus are wild vectors of *T. cruzi* in Chile. *Mepraia* specimens infected with *T. cruzi* have been detected in Pan de Azúcar and Santa María islands. The most common vertebrates that inhabit these islands are birds and reptiles, and it is unknown whether small mammals are present. Consequently, it is relevant to know whether there are any *T. cruzi*-infected small mammals on those islands to elucidate the *T. cruzi* cycle. To clarify this crossroads, islands of northern Chile were explored to determine if *T. cruzi*-infected triatomines and rodents co-occur in islands of northern Chile. *T. cruzi* DNA was detected by conventional and real-time PCR in three islands: on Santa María and Pan de Azúcar islands* T. cruzi* was detected in *Mepraia* sp samples, while on Pan de Azúcar (6.1%) and Damas islands (15%) was detected in the rodent *Abrothrix olivacea*. We show for the first time in Chile the occurrence of insular rodents infected with *T. cruzi*, and a complete *T. cruzi* life cycle in a coastal island. Our results provide new insights to understand the *T. cruzi* infection in the wild cycle.

## Introduction

*Trypanosoma cruzi* is the cause agent of Chagas disease, one of the main zoonotic diseases mediated by vectors in America. This parasite is transmitted principally by blood-feeding bugs of the subfamily Triatominae. The *T. cruzi* life cycle circulates among triatomines and several mammalian host species while birds and reptiles still are considered refractory to *T. cruzi* infection (Kierszenbaum, Ivanyi & Budzko, 1976; Urdaneta-Morales & McLure, 1981). *Mepraia* is a genus (Mazza, Gajardo & Jörg, 1940) of Triatominae endemic to arid and semiarid regions of Chile; it plays an important role in the wild cycle of *T. cruzi* transmission, and its species are potential vectors for humans (Botto-Mahan et al., 2008; Campos-Soto et al., 2016).

Three species are currently included in the genus *Mepraia*: *M. gajardoi*, *M. parapatrica* and *M. spinolai* (Frías, Henry & Gonzalez, 1998; Frías-Lasserre, 2010). The first two inhabit coastal areas, while *M. spinolai* inhabits coastal and interior valleys. *M. parapatrica* is distributed in the coastal desert in an area intermediate between those of *M. spinolai* and *M. gajardoi* (Frías-Lasserre, 2010; Campos et al., 2013). Island populations of *M. parapatrica* have been reported inhabiting Pan de Azúcar Island in the Atacama Region (Sagua et al., 2000; Frías-Lasserre, 2010). Sagua et al. (2000) suggested that triatomines from Pan de Azúcar Island feed mainly on seabirds (78%), marine mammals (15%) and reptiles (7%). Individuals of *Mepraia* sp. were also reported in Santa María Island in the Antofagasta Region (Rives-Blanchard et al., 2017).

The presence of triatomines infected with *T. cruzi,* as well as mixed infections with more than one *T. cruzi* Discrete Typing Unit (DTU), were reported in both islands (Rives-Blanchard et al., 2017). Mixed infections are more common in ecotopes with high infection rate or high diversity of mammals that harbor different *T. cruzi* lineages (Campos-Soto et al., 2016). However, the most frequent vertebrates that inhabit both islands are lizards, seabirds and few marine mammals such as seawolf, sea otters and migratory cetaceans (R. Campos-Soto, 2018, field observations of this study). Small mammals inhabiting these islands are unknown, despite their potential major role in the *T. cruzi* life cycle. Given that there are two islands with infected bugs, are there any *T. cruzi*-infected small mammals on those islands? Therefore, sampling insular triatomines and small mammals as possible reservoirs of *T. cruzi* in coastal islands of northern Chile is key to clarify this question and the *T. cruzi* life cycle. We studied two major islands in the north of Chile (Pan de Azúcar and Santa María) together with three additional islands in which hosts of *T. cruzi* are unknown without previous infection studies. The evidence provided by this study offers new opportunities to examine the *T. cruzi* life cycle in coastal islands of northern Chile.

## Materials & Methods

### Areas of small mammal and triatomine collections

**Figure 1:**
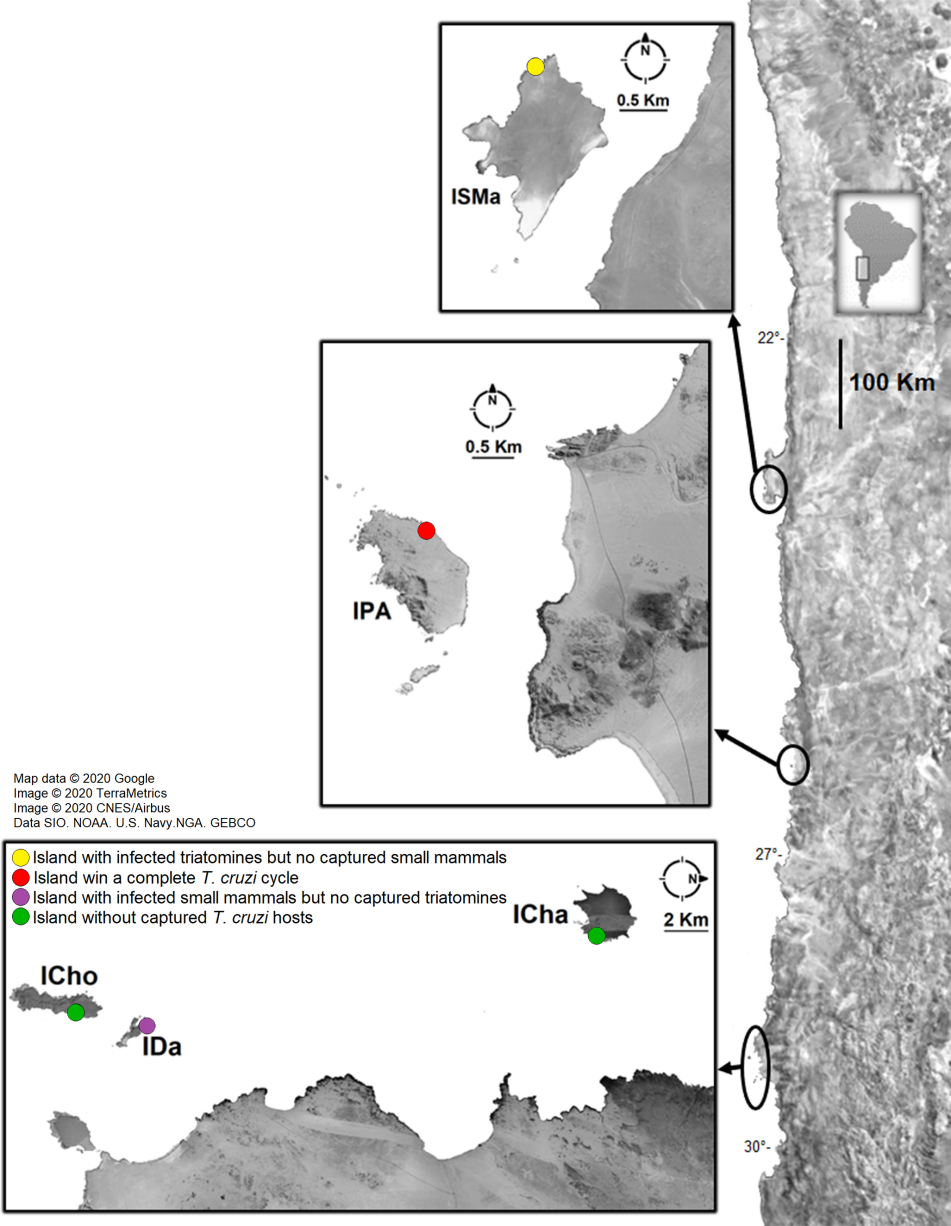
Sample localities of small mammal and triatomine collection. ISMa: Santa María Island, IPA: Pan de Azúcar Island, ICha: Chañaral Island, ICho: Choros Island, IDa: Damas Island. Attributions: Map data©2020 Google, Image ©2020 TerraMetrics, Image©2020 CNES/Airbus, Data SIO. NOAA. US Navy. NGA. GEBCO. Map was modified by illustrations purposes.

Small mammals and *Mepraia* individuals were collected during the summer (2017 to 2019) in five coastal islands of northern Chile: Santa María Island (distant site 1.9 km from the continent), in Antofagasta Region; Pan de Azúcar Island (distant site 1.8 km from the continent), in Pan de Azúcar National Park, Atacama Region; Chañaral Island (distant site 8.5 km from the continent), in Atacama Region; Damas Island (in a site at 5.5 km from the continent) and Choros Island (distant site 8.6 km from the continent) in Coquimbo Region. The last three islands are included in the Pingüino de Humboldt National Park. Localities and their geographical locations are shown in Fig. 1 and Table 1. Only on Chañaral Island two field activities were carried out.

**Table 1:** Collection sites in islands and *Trypanosoma cruzi* infections in small mammals and triatomines by kDNA conventional PCR and satDNA real-time PCR.

**Island**	**Latitude/longitude**	**Host species**	**ID Host**	**kDNA PCR**	**SatDNA qPCR**
Santa María	23°25′51″S/70°36′31″W	Smam. not found	–	–	–
		Triat. *Mepraia* sp	1isma	+	(-)
			2isma	+	+
			3isma	+	+
			4isma	+	+
			5isma	(-)	+
			7isma	(-)	+
			9isma	+	+
			10isma	+	(-)
			12isma	+	+
			14isma	+	+
			16isma	+	+
			18isma	(-)	+
			22isma	+	+
			23isma	+	+
			26isma	(-)	+
			27isma	(-)	+
			Inf. rate	11/38 (28.9%)	14/38 (36.8%)
Pan de Azúcar	26°9′6″S/70°41′7″W	Smam. *Abrothrix olivaceus*	8Aipa	+	+
			25Aipa	+	+
			Inf. rate	2/33 (6.1%)	2/33 (6.1%)
		Triat. *Mepraia parapatrica*	1ipa	+	+
			2ipa	+	+
			5ipa	(-)	+
			6ipa	+	+
			7ipa	+	(-)
			8ipa	(-)	+
			9ipa	(-)	+
			10ipa	(-)	+
			15ipa	(-)	+
			17ipa	(-)	+
			19ipa	(-)	+
			23ipa	(-)	+
			26ipa	(-)	+
			29ipa	+	(-)
			40ipa	+	(-)
			Inf. rate	6/59 (10.17%)	12/59 (20%)
Chañaral	29°2′17″S/71°34′8″W	Smam. *Thylamys elegans*		0/9	0/7
		Triat. not found	–	–	–
Damas	29°13′49″S/71°31′47″W	Smam. *Abrothrix olivaceus*	12ida	+	+
			18ida	+	+
			21ida	+	+
			Inf. rate	3/20 (15%)	3/20 (15%)
		Triat. not found	–	–	–
Choros	29°2′17″S/71°34′8″W	Smam. not found	–	–	–
		Triat. not found	–	–	–

### Small mammal and triatomine sampling

Insular rodents were caught with standard Sherman traps (8 × 9 × 23 cm). Trapping effort was 100 traps/night and conducted for two nights at each site. This sampling design was previously shown successful to capture small mammals (Boric-Bargetto et al., 2016). A mixture of oats (900 grs) and vanilla essence (150 ml) was used as bait, which have been used in several studies with efficient results (Torres-Pérez et al., 2004; Boric-Bargetto et al., 2012). Small mammals were anesthetized in the field with isoflurane and 0.2 ml of blood was sampled in a field laboratory. Fresh blood samples were received in a cryotube and conserved in a liquid nitrogen container. Blood samples were taken only from adult rodents; juvenile and pregnant females were released. The captured rodents were marked with a temporal nontoxic-highlighter and released once they were well awake and recovered. Triatomines were collected passively as described in Campos-Soto, Torres-Pérez & Solari (2015) by qualified personnel, when this method was unsuccessful triatomines were collected actively by lifting stones in rock piles and nests. Captured insects were transported to the laboratory and maintained in a climate chamber at 27 ° C with a relative humidity of 50% and a 14:10 h light:dark photoperiod. Then the complete gut of triatomines was dissected and used for DNA extraction.

### Ethics statement

All individuals were manipulated following the standard bioethics and biosafety protocols proposed by the American Society of Mammalogists (Sikes, 2016). Sampling procedures were authorized by the Servicio Agrícola y Ganadero (resolution number: 8353), Corporación Nacional Forestal from Atacama Region (permit number: 049/2017) and Coquimbo Region (permit number: 22/2019). We appreciate the logistical help was provided by Pan de Azúcar National Park and Pingüino de Humboldt National Reserve administrators and their park rangers. The research project that includes this study was approved by the Bioethic and Biosecurity Committee of the Pontificia Universidad Católica de Valparaíso (permit number: BIOEPUCV-A98b-2017).

### DNA extraction from triatomines and blood of small mammals

DNA was extracted from blood samples and intestinal contents of triatomines using the DNeasy^®^ Blood & Tissue kit (QIAGEN). The protocol was carried out according to the manufacturer’s instructions; the DNA was eluted twice with 100 µL of elution buffer. All samples were co-extracted with 100 pg of a sequence of *Arabidopsis thaliana* used as a heterologous internal amplification control (IAC) as previously described in Mc Cabe et al. (2019) to discount loss of DNA or carryover of polymerase chain reaction (PCR) inhibitors.

### Kinetoplast DNA conventional PCR assays

Assays were performed for all samples using kinetoplast DNA (kDNA) primers 121 (AAATAATGTACGGGKGAGATGCATGA) and 122 (GGTTCGATTGGGGTTGGTGTAATATA) (Wincker et al., 1994) and the polymerase fast PCR Master Mix SapphireAmp^®^ (Takara). Cycling conditions were 30 s at 94 °C, followed by 40 cycles at 94 °C for 30 s, 55 °C for 30 s and 72 °C for 1 min according to Takara manufacturer’s instructions in a Bioer model TC-96/G/H(b)C LifeEco^®^ thermocycler. Verification of amplification of a variable region of 330 bp of minicircle kDNA was assessed by 2% agarose gel electrophoresis. Each sample was tested twice to confirm the infection with *T. cruzi;* the sample was considered infected with *T. cruzi* when at least one of the two amplifications resulted positive.

### Satellite DNA real-time PCR assays

Assays were performed using *T. cruzi* nuclear satellite DNA (satDNA) primers Cruzi 1 (ASTCGGCTGATCGTTTTCGA) and Cruzi 2 (AATTCCTCCAAGCAGCGGATA) (Piron et al., 2007) in a final volume of 20 µL containing 5 µL DNA template as previously described in Muñoz-San Martín et al. (2020). Each sample was tested in duplicate.

### Parasite standard calibration curve

*T. cruzi* DNA standards for absolute quantification were obtained from 10^5^ parasite equivalents/mL (par-eq/mL) of clonal reference strains Dm28c (TcId) and Y (TcII) and 10-fold serial dilutions were performed with nuclease-free water (range between 10^5^ and 10^1^ par-eq/mL) as previously described in Muñoz-San Martín et al. (2020).

### Heterologous internal amplification control qPCR Assays

Assays were performed in blood samples using primers IAC Fw (5′ACCGTCATGGAACAG CACGTA 3′) and IAC Rv (5′ CTCCCGCAACAAACCCTATAAAT 3′) Duffy et al., 2013 at a final concentration of 0.2 µM and at a melting temperature of 58 °C as previously described in (Mc Cabe et al., 2019). Quantification of the parasite equivalents from DNA samples was calculated considering the amplification curve of standard *T. cruzi* DNA and the results were normalized according to the heterologous IAC results.

### Genotyping assays

Four DTU real-time PCR genotyping assays were performed for mammal samples positive for *T. cruzi* (Muñoz Martín, Apt & Zulantay , 2017). Detection of TcI, TcII, TcV, and TcVI was performed using the same primers, concentrations, and controls as previously described in (Muñoz-San Martín et al., 2018). The other assay conditions, including the cycling profile, are described above. Controls were always included in each reaction and each sample was tested in duplicate.

## Results

### Small mammal and triatomine collection

**Figure 2:**
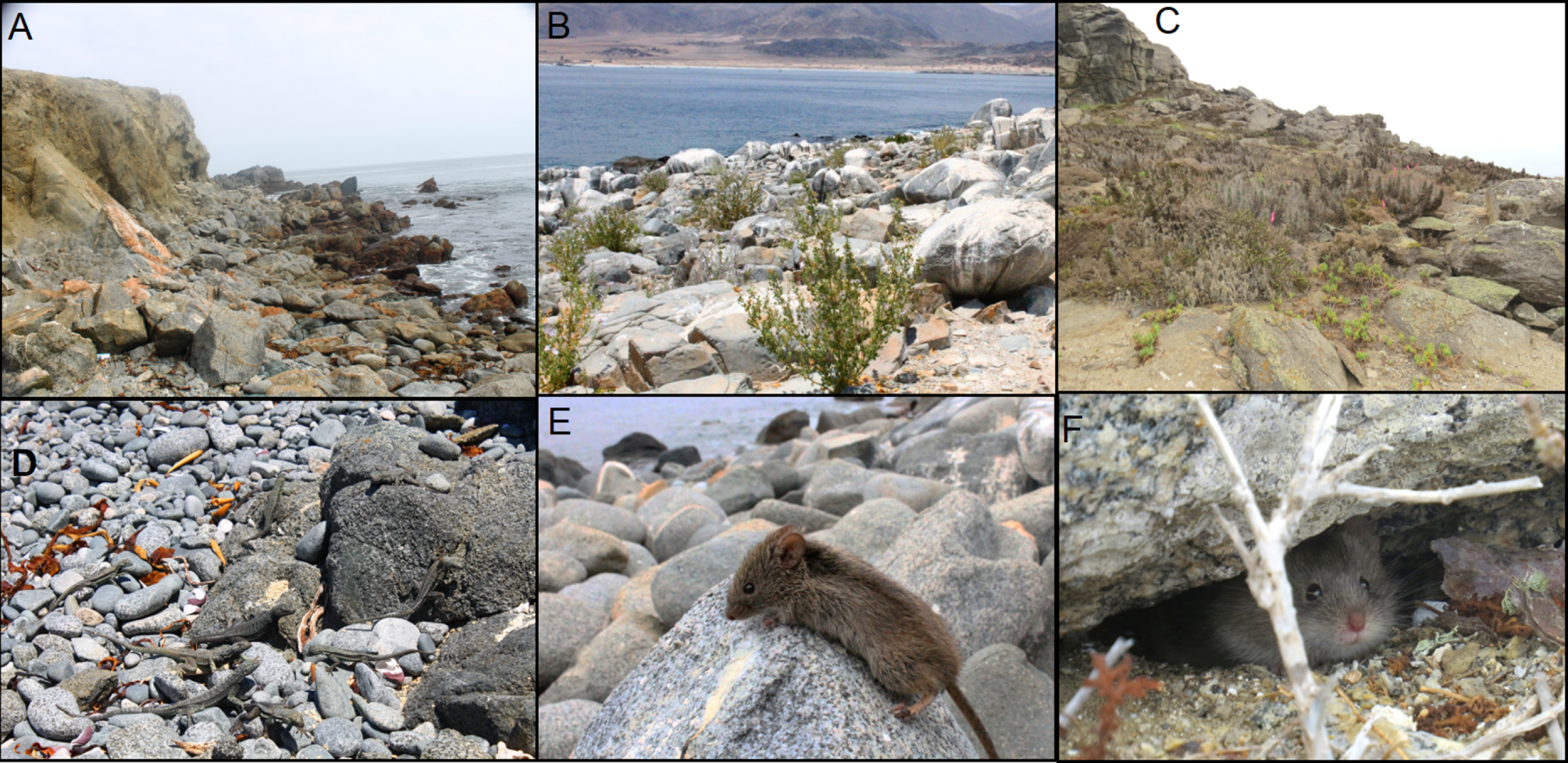
Sampling sites on islands with detected *Trypanosoma cruzi* hosts. (A) Santa María Island. (B) Pan de Azúcar Island. (C) Damas Island. (D) high abundance of *Microluphus atacamensis* on Santa María Island. (E) *Abrothrix olivacea* form Pan de Azúcar Island. (F) *A. olivacea* form Damas Island. Photos A, B, D and E credit: Ricardo Campos-Soto, photos C and F credit: Javier Cruz.

Small mammals were captured in three islands (Figs. 1 and 2, Table 1). In Pan de Azúcar Island, 49 *Abrothrix olivacea* (Sigmodontinae) were captured in one sampling night; 33 blood samples were obtained. In Chañaral Island, nine mouse opossums of the species *Thylamys elegans* (Didelphidae) were captured in two sampling nights in the first field activity (Fig. S1), obtaining nine blood samples. In Damas Island, 48 *Abrothrix olivacea* were captured in two sampling nights, obtaining 20 blood samples (Table 1, Fig. 2). No small mammals were found in Santa María and Choros Islands. *Mepraia* triatomines were captured in Santa María Island (*N* = 38) and Pan de Azúcar Island (*N* = 59); no triatomines were found in the other three islands. Collected small mammals and triatomines for each island and their geographical coordinates are detailed in Table 1 and Fig. 1.

### Kinetoplast DNA conventional PCR assays

**Figure 3:**
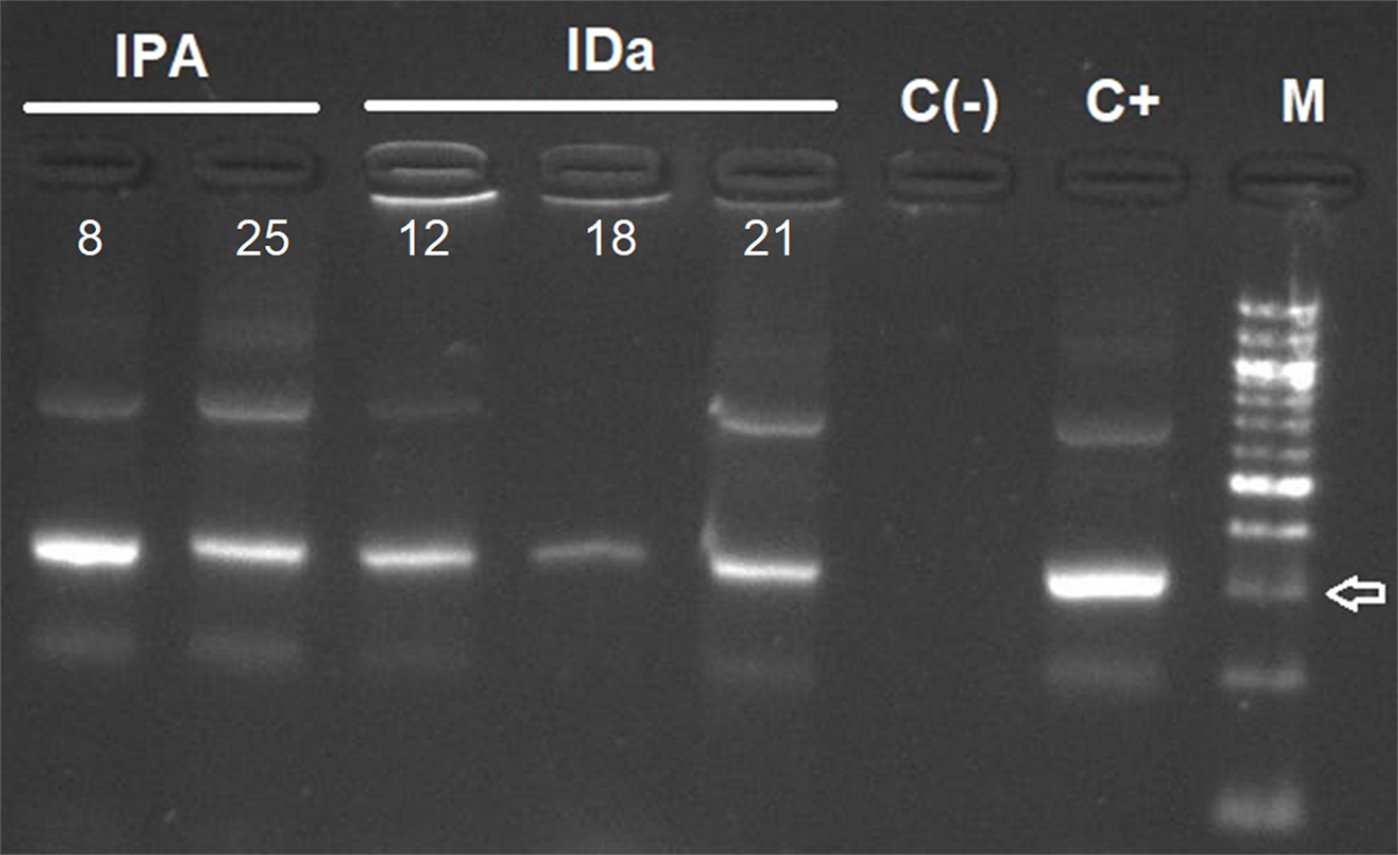
Agarose gel electrophoresis of amplified kDNA by conventional PCR in small mammals (*Abrothrix olivacea*). Lanes IPA: positive rodents from Pan de Azúcar Island, IDa: positive rodents from Damas Island. Lanes C(-): negative control, C+: Positive control and M: 100-bp DNA ladder. Arrow indicates 300 bp. Photo credit: Gabriel Díaz-Campusano.

*T. cruzi* kDNA in small mammals was detected in two *Abrothrix olivacea* in Pan de Azúcar Island, representing an infection rate of 6.06%. No infected *Thylamys elegans* were found in Chañaral Island, and three *A. olivacea* were detected with *T. cruzi* (infection rate of 15%) in Damas Island (Table 1, Fig. 3). For triatomines, 11 samples of *Mepraia* were positive for *T. cruzi* in Santa María Island, representing an infection rate of 28.9% (Fig. S2, Table 1). In Pan de Azúcar Island we detected six samples of *Mepraia* positive for *T. cruzi*, representing an infection rate of 10.17% (Fig. S3, Table 1).

### Satellite DNA real-time PCR assays

For *T. cruzi* satDNA detection in small mammals, all the same *A. olivacea* samples were positive as in the detection by kDNA (Table 1, Fig. S4). The genomic quantification by real-time PCR only was possible for *Abrothrix* from Pan de Azúcar Island, with 129 (sample 8Aipa) and 3.6 (sample 25Aipa) par-eq/mL while in *Abrothrix* from Damas Island low parasitemias were detected (<1 par-eq/mL). For *T. cruzi* satDNA detection in triatomines, 14 *Mepraia* samples for Santa María Island were positive, representing an infection rate of 36.8%, while in Pan de Azúcar Island we found 12 positive samples, with an infection rate of 20.33% (Table 1, Fig. S4). Raw data of real-time PCR analyses in small mammals and triatomines are available in Table S1.

### Genotyping assays

Genotyping *T. cruzi* DTU assays were only performed in the *Abrothrix* from Pan de Azúcar Island because higher parasitemia was detected. We found the TcII DTU in a mixed infection with TcVI in *A. olivacea* (sample 8Aipa), and a single infection with TcII (sample 25Aipa).

## Discussion

The wild cycle of *T. cruzi* in continental areas has been widely reported, but there is still remains a lack of knowledge in insular areas. A few examples were reported that revealed the enzootic *T. cruzi* cycle that included mammals and triatomines in islands of Brazil (Grisard et al., 2000; Das Xavier et al., 2014). In Chile, *T. cruzi-* infected triatomines were reported in Santa María and Pan de Azúcar islands (Rives-Blanchard et al., 2017), raising question related to the mammal hosts involved in this *T. cruzi* cycle.

On Pan de Azúcar Island, two *A. olivacea* were positive to *T. cruzi* by kDNA PCR and satDNA qPCR (infection rate 6.06%, Table 1, Fig. 3). It has been suggested that triatomines on Pan de Azúcar Island feed mainly on sea birds (Sagua et al., 2000), which may explain the low infection rate found in *A. olivacea*. Interestingly, one of the *A. olivacea* samples showed a mixed infection with two DTU, TcI and TcII, congruent with the two DTUs previously found in *Mepraia* on Pan de Azúcar Island (Rives-Blanchard et al., 2017). Therefore, our results confirm that on Pan de Azúcar Island there is a complete *T. cruzi* life cycle, i.e., a cycle in which *T. cruzi* circulates through triatomines and mammalian blood.

Three *A. olivacea* on Damas Island resulted positive for *T. cruzi* by kDNA PCR and satDNA qPCR (infection rate 15% Table 1, Fig. 3). However, no triatomines were found in our study, a pattern also detected on Chañaral and Choros islands. It has been reported that many triatomines foci can go undetected when vector density is low (Abad-Franch et al., 2014). A likely explanation is that we failed to find triatomines on those islands particularly if they occur at low abundance (sampling bias). However, we sampled intensively using both a passive (Campos-Soto, Torres-Pérez & Solari, 2015) and active method, and the park rangers never reported seeing triatomines (personal communication with park rangers). A study suggests that the current absence of *M. spinolai* in those islands may be explained by their absence when the islands were formed or that ancient allopatric populations were extinguished (Campos-Soto et al., 2020). If hypothetically triatomines are not present, alternatives to explain the *T. cruzi* infection in rodents include: (i) Maintenance of the parasite by vertical trans-placental transmission between rodents, which has been reported in humans, bats and mice (Delgado & Santos-Buch, 1978; Añez, Crisante & Soriano, 2009; Ortiz et al., 2012); (ii) Cross-reaction with other trypanosomatids (see below).

On Santa María Island, individuals of *Mepraia* infected with *T. cruzi* were previously reported (Rives-Blanchard et al., 2017), but no small mammals were found in our study despite the sampling effort was similar to that performed on Pan de Azúcar and Damas islands. Unlike the other islands, Santa María Island lacks of vegetation (Fig. 2, Fig. S1), and the influence of climatic conditions create an arid and desertic landscape (Jerez, 2000; Cavieres et al., 2002; Clarke, 2006). Also, this island had very few suitable places to set traps (Fig. 2). Under these conditions, detecting small mammals can be difficult, likely impacting our results. Future studies including higher sampling effort both in density and temporal may elucidate this finding. Strikingly, we found triatomines with an infection rate of 28.9% by kDNA PCR and 36.8% by satDNA qPCR (23.6% confirmed by both, Table 1). These values reveal a high infection rate, particularly taking into account that the most abundant vertebrates inhabiting this island are marine birds and reptiles of the genus *Microlophus* (Fig. 2; R. Campos-Soto, 2018, field observations of this study).

Mixed infection was previously reported in bugs from Santa María Island (Rives-Blanchard et al., 2017). It has been suggested that mixed infections are more frequent in areas with high infection rate and/or there is high diversity of mammals that harbor different *T. cruzi* lineages (Campos-Soto et al., 2016). This is congruent with our triatomine infection rates but contrasts with our small mammals captures. Despite we cannot confirm the absence of small mammals on Santa María Island, the question of how the *T. cruzi* cycle is maintained still remains. In the absence of small mammals on Santa María Island, one explanation for our results is the horizontal transmission of *T. cruzi* among triatomines. For example, coprophagy and cleptohematophagy were reported as (uncommon) mechanisms of transmission among triatomine vectors, mainly by young nymphs (Ryckman, 1951; Schaub, 1988). On the other hand, Rives-Blanchard et al. (2017) showed that there are positive triatomines on Santa María Island, in which *T. cruzi* DTUs were not identified by hybridization assays. The authors suggested that some TcI or TcII variants did not hybridize with the probes used, or that there are other DTUs not analyzed in their study. Alternatively, there is the possibility that these unidentified DTUs could be another *Trypanosoma* with cross-reactivity to *T. cruzi*. In fact, cross-reactivity in parasite detection by PCR and qPCR analyses has been reported between *T. cruzi* and *T. rangeli* (Ramírez et al., 2015; Seiringer et al., 2017). The only triatomines that can transmit *T. rangeli* are *Rhodnius* and *Panstrongylus* (Vallejo et al., 2015). These genera do not co-occur with *Mepraia,* therefore cross-reactivity with *T. rangeli* is unlikely*.*

Lizard and avian trypanosomes phylogenetically related to *T. cruzi* has been reported (Hughes & Piontkivska, 2003; Hamilton, Gibson & Stevens, 2007; Viola et al., 2008; Dario et al., 2017). Reptiles of the genus *Microlophus* and marine birds are found in high abundance on Santa María Island (Fig. 2), thus triatomines may feed mainly of bird and reptile blood. Also, it has been observed that *Microlophus* actively hunt and feed on these triatomines (R. Campos-Soto, 2018, field observations of this study). Consequently, the triatomines could be hosting a reptilian or avian trypanosome that could have cross-reactivity with *T. cruzi.* However, reptilian trypanosomes are transmitted by dipterous sandflies and not by triatomines (Adler & Theodor, 1957; Hamilton, Gibson & Stevens, 2007). According to Seiringer et al. (2017), the best *T. cruzi* diagnosis is a combination of both kinetoplast DNA detection and nuclear satellite DNA by conventional PCR and qPCR assays, respectively. In our study, all small mammals and most of the triatomines were positive for *T. cruzi* by conventional PCR (targeting kDNA) and qPCR (targeting nuclear satellite DNA), suggesting the absence of cross-reaction with reptilian or avian trypanosomes. Trypanosomes such as *T. brucei* can exceptionally infect lizards in the wild cycle (Njagu et al., 1999), and chickens experimentally (Minter-Goedbloed, 1981). A study shows that the availability of reptiles is positively related to the *T. cruzi* infection risk in an endemic area of Chile (San Juan et al., 2020). These antecedents show that the role of endemic reptiles as hosts of *T. cruzi* remains to be elucidated*.*

The mechanisms of colonization of *Mepraia* to the islands of northern Chile are relevant to understand the *T. cruzi* infection in these areas. The origin of *Mepraia* populations on Santa María and Pan de Azúcar islands was suggested by mechanisms of vicariance and dispersal, starting about middle-upper Pleistocene. Bidirectional migration rates between these islands and continental populations was inferred (Campos-Soto et al., 2020). Possible means of dispersal include passive transport by marine birds (Schofield et al., 1998; Sagua et al., 2000) and fishermen who sail to the islands carrying infected triatomines and/or eggs in their clothes or backpacks. An additional passive dispersal mechanism might include sea wolves (*Otaria flavescens*), which could transport nymphs within their pelage (Schofield et al., 1998). The flight of kissing-bugs also may be another dispersal mechanism. However, *Mepraia*’s nymphs and adult females are wingless while males show wings polymorphism (Schofield et al., 1998; Campos et al., 2011). *M. parapatrica* and *M. gajardoi* males are brachypterous (Frías-Lasserre, 2010), with wings shorter or equal than the abdomen length (Campos et al., 2011) and flying capacity not documented, which would allow discard the dispersion by flight of the bugs.

## Conclusions

In conclusion, we show for the first time in Chile the occurrence of insular rodents infected with *T. cruzi*, and a complete *T. cruzi* life cycle in a coastal island (Pan de Azúcar Island). We also show two different contrasting results: an island (Santa María) with infected triatomines but without captured small mammals, and another island (Damas) with infected rodents but without captured triatomines (Fig. 1, Table 1). Future studies including a greater capture effort targeting the hosts and the vector will help to elucidate the transmission mechanism maintaining the *T. cruzi* life cycle on those islands. Our study provides new relevant knowledge about the *T. cruzi* cycle on islands and the role of its hosts and vectors.

##  Supplemental Information

10.7717/peerj.9967/supp-1 Supplemental Information 1 Island without detected *Trypanosoma cruzi* hosts (A) Chañaral Island. (B) *Thylamys elegans* from** Chañaral Island. (C) Choros Island. Photo credits: Ricardo Campos-Soto.

10.7717/peerj.9967/supp-2 Supplemental Information 2 Agarose gel electrophoresis of amplified kDNA by conventional PCR in triatomines from Santa María Island Lanes C(-): negative control, C+: Positive control and M: 100-bp DNA ladder. Arrow indicates 300 bp. Photo credit: Gabriel Díaz-Campusano.

10.7717/peerj.9967/supp-3 Supplemental Information 3 Agarose gel electrophoresis of amplified kDNA by conventional PCR in triatomines from Pan de Azúcar Island Lanes C(-): negative control, C+: Positive control and *: indicates positive sample. Photo credit: Nicol Quiroga.

10.7717/peerj.9967/supp-4 Supplemental Information 4 Cycle threshold (Ct) values of positive Satellite DNA real-time PCR assays in small mammals and triatomines from islands (A) Blood samples of *Abrothrix olivacea*. (B) Intestinal contents samples of *Mepraia* sp. The line inside the box represents the median, and the box extends from the lower to the upper quartiles. Whiskers indicate min to max and dots represent the samples. Figure credit: Catalina Muños San-Martín.

10.7717/peerj.9967/supp-5 Supplemental Information 5 Raw data result of qPCR analyses in small mammals and triatomines The *T. cruzi* column refers to the result of satellite DNA amplification; the Ct is the cycle number at which the fluorescence generated within a reaction crosses the threshold, the par-eq/mL is the absolute quantification result. The IAC column shows the result of a sequence of *Arabidopsis thaliana* DNA amplification. Normalization: IAC results were used for normalization of the quantification result (small mammals only).

10.7717/peerj.9967/supp-6 Supplemental Information 6 Raw data of real-time PCR analyses

## Additional Information and Declarations

### Field Study Permissions

The following information was supplied relating to field study approvals (i.e., approving body and any reference numbers): Sampling procedures were authorized by the Servicio Agrícola y Ganadero (resolution number: 8353), Corporación Nacional Forestal from Atacama Region (permit number: 049/2017) and Coquimbo Region (permit number: 22/2019). We appreciate the logistical help was provided by Pan de Azúcar National Park and Pingüino de Humboldt National Reserve administrators and their park rangers.

### Data Availability

The following information was supplied regarding data availability: The raw measurements are available in the Supplementary Files.
