# Supplementary figures and images for: Trypanosoma cruzi-infected triatomines and rodents co-occur in a coastal island of northern Chile
Source: PeerJ. 2020 Oct 14;8:e9967. doi: 10.7717/peerj.9967 (PMC7568477; doi:10.7717/peerj.9967)

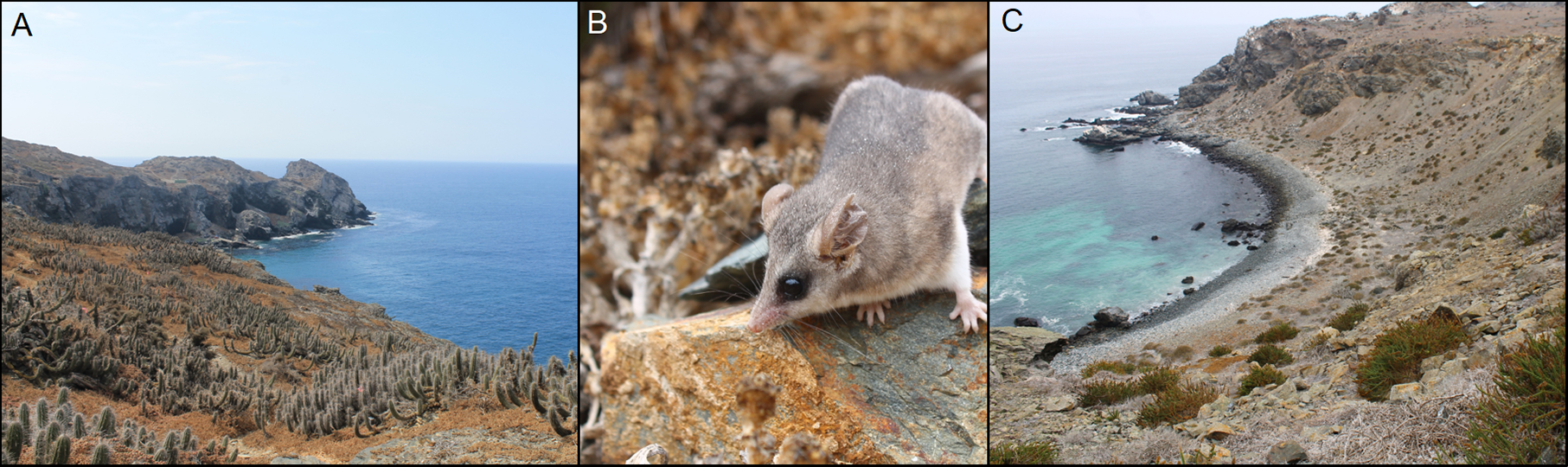

Supplement: Supplemental Information 1 — (A) Chañaral Island. (B) Thylamys elegans from Chañaral Island. (C) Choros Island. Photo credits: Ricardo Campos-Soto. [file peerj-08-9967-s001.png]

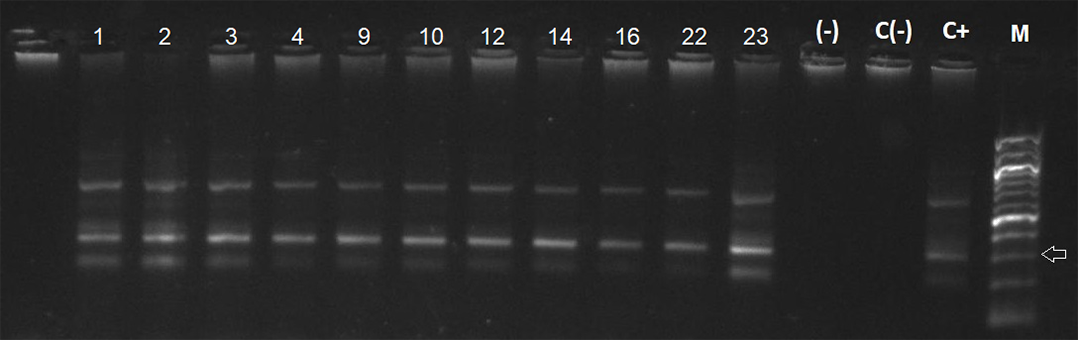

Supplement: Supplemental Information 2 — Lanes C(-): negative control, C+: Positive control and M: 100-bp DNA ladder. Arrow indicates 300 bp. Photo credit: Gabriel Díaz-Campusano. [file peerj-08-9967-s002.png]

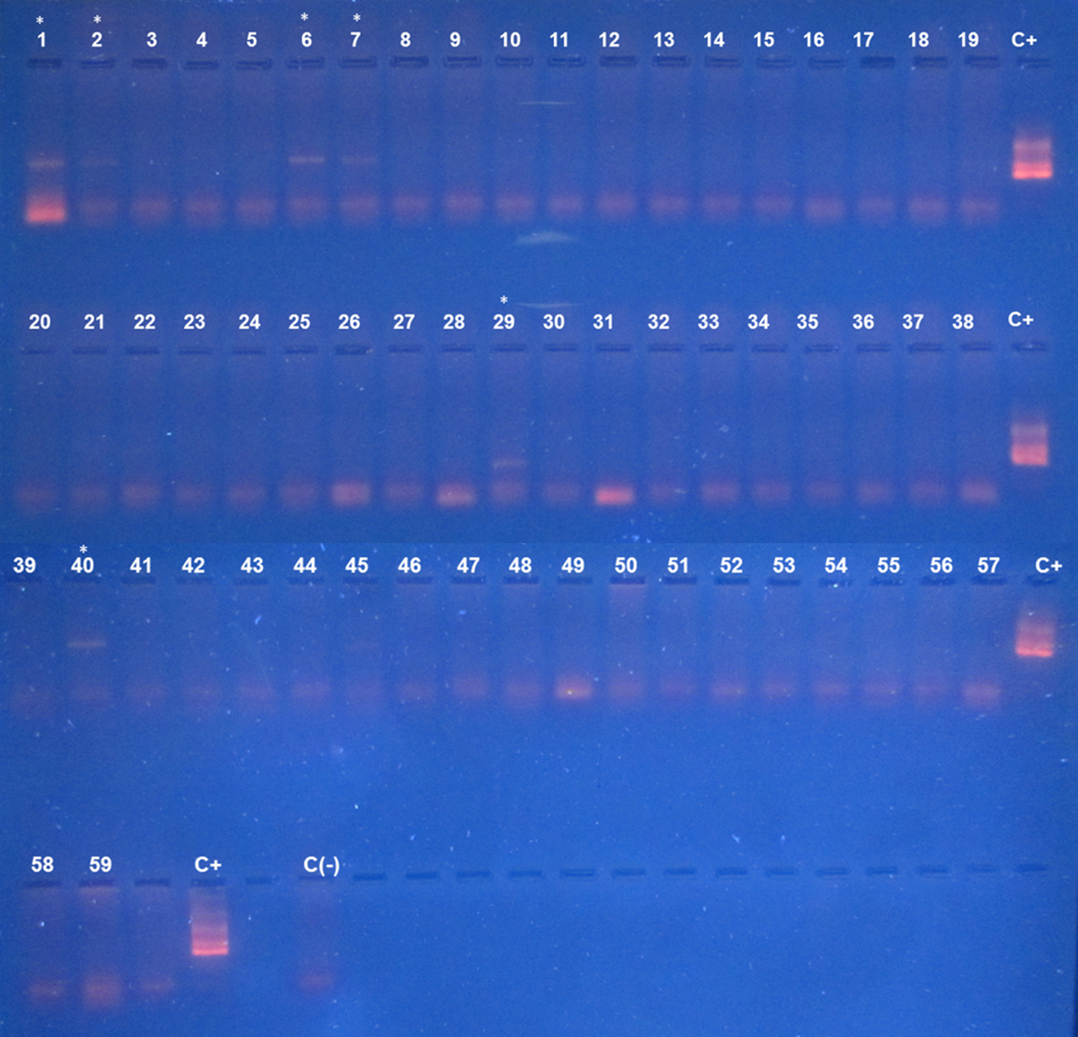

Supplement: Supplemental Information 3 — Lanes C(-): negative control, C+: Positive control and *: indicates positive sample. Photo credit: Nicol Quiroga. [file peerj-08-9967-s003.png]

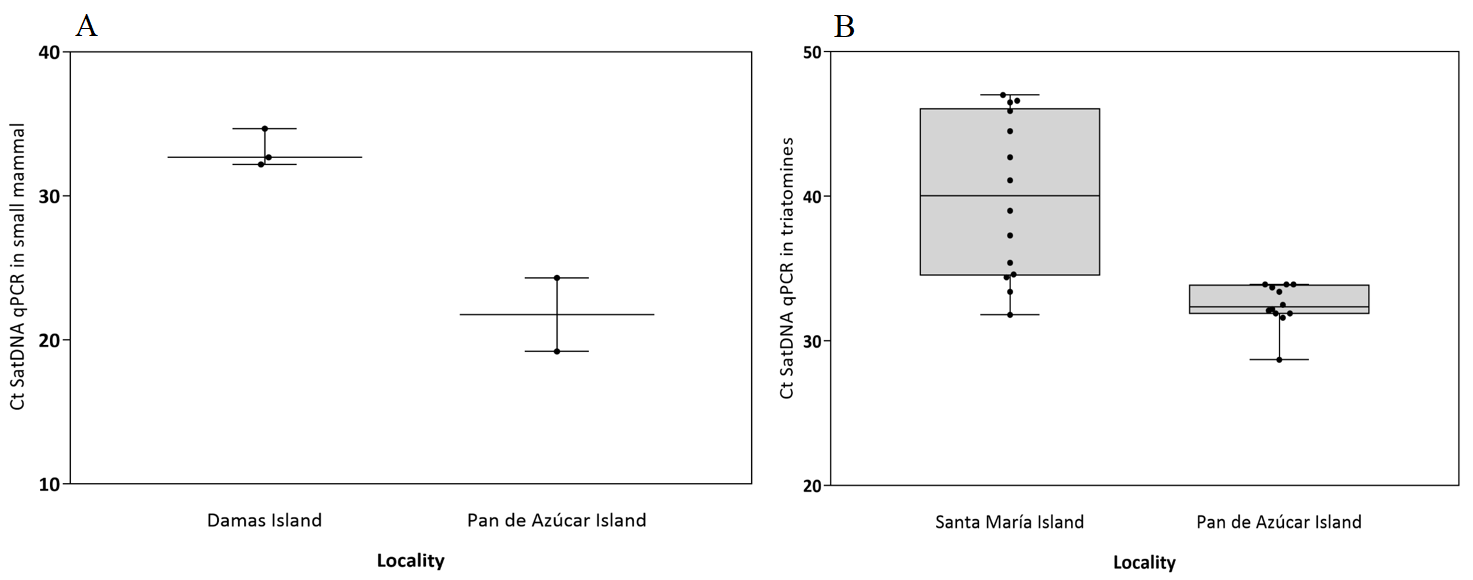

Supplement: Supplemental Information 4 — (A) Blood samples of Abrothrix olivacea. (B) Intestinal contents samples of Mepraia sp. The line inside the box represents the median, and the box extends from the lower to the upper quartiles. Whiskers indicate min to max and dots represent the samples. Figure credit: Catalina Muños San-Martín. [file peerj-08-9967-s004.png]
