# Supplementary material for: Trypanosoma cruzi-infected triatomines and rodents co-occur in a coastal island of northern Chile
Source: PeerJ. 2020 Oct 14;8:e9967. doi: 10.7717/peerj.9967 (PMC7568477; doi:10.7717/peerj.9967)
Supplement: Supplemental Information 5 — The T. cruzi column refers to the result of satellite DNA amplification; the Ct is the cycle number at which the fluorescence generated within a reaction crosses the threshold, the par-eq/mL is the absolute quantification result. The IAC column shows the result of a sequence of Arabidopsis thaliana DNA amplification. Normalization: IAC results were used for normalization of the quantification result (small mammals only). [file peerj-08-9967-s005.docx]

|  |  |  | **Amplification by real-time PCR** | | | |  |
| --- | --- | --- | --- | --- | --- | --- | --- |
|  |  |  | ***T. cruzi*** | | **IAC** | | **Normalization** |
| **Island** | **Species** | ID | Ct | par-eq/mL | Ct | pg/uL | par-eq/mL |
| Pan de Azúcar | *Abrothrix olivaceus* | 1 | no Ct | 0,00 | 19,55 | 0,119 | 0 |
| Pan de Azúcar | *Abrothrix olivaceus* | 2 | no Ct | 0,00 | 19,54 | 0,120 | 0 |
| Pan de Azúcar | *Abrothrix olivaceus* | 3 | no Ct | 0,00 | 21,23 | 0,038 | 0 |
| Pan de Azúcar | *Abrothrix olivaceus* | 5 | no Ct | 0,00 | no Ct | 0,000 | 0 |
| Pan de Azúcar | *Abrothrix olivaceus* | 7 | no Ct | 0,00 | 20,63 | 0,057 | 0 |
| Pan de Azúcar | *Abrothrix olivaceus* | 8 | 19,21 | 57,77 | 17,60 | 0,448 | 129,0 |
| Pan de Azúcar | *Abrothrix olivaceus* | 9 | no Ct | 0,00 | 17,58 | 0,454 | 0 |
| Pan de Azúcar | *Abrothrix olivaceus* | 13 | no Ct | 0,00 | 17,16 | 0,599 | 0 |
| Pan de Azúcar | *Abrothrix olivaceus* | 15 | no Ct | 0,00 | 21,47 | 0,033 | 0 |
| Pan de Azúcar | *Abrothrix olivaceus* | 16 | no Ct | 0,00 | 22,08 | 0,021 | 0 |
| Pan de Azúcar | *Abrothrix olivaceus* | 18 | no Ct | 0,00 | 26,41 | 0,001 | 0 |
| Pan de Azúcar | *Abrothrix olivaceus* | 19 | no Ct | 0,00 | 19,05 | 0,167 | 0 |
| Pan de Azúcar | *Abrothrix olivaceus* | 20 | no Ct | 0,00 | 24,45 | 0,004 | 0 |
| Pan de Azúcar | *Abrothrix olivaceus* | 21 | no Ct | 0,00 | 20,75 | 0,053 | 0 |
| Pan de Azúcar | *Abrothrix olivaceus* | 22 | no Ct | 0,00 | 23,08 | 0,011 | 0 |
| Pan de Azúcar | *Abrothrix olivaceus* | 23 | no Ct | 0,00 | 19,63 | 0,113 | 0 |
| Pan de Azúcar | *Abrothrix olivaceus* | 25 | 24,31 | 1,68 | 17,53 | 0,468 | 3,6 |
| Pan de Azúcar | *Abrothrix olivaceus* | 26 | no Ct | 0,00 | 18,65 | 0,204 | 0 |
| Pan de Azúcar | *Abrothrix olivaceus* | 27 | no Ct | 0,00 | 17,69 | 0,390 | 0 |
| Pan de Azúcar | *Abrothrix olivaceus* | 28 | no Ct | 0,00 | 21,32 | 0,033 | 0 |
| Pan de Azúcar | *Abrothrix olivaceus* | 29 | no Ct | 0,00 | 20,40 | 0,063 | 0 |
| Pan de Azúcar | *Abrothrix olivaceus* | 30 | no Ct | 0,00 | 17,13 | 0,572 | 0 |
| Pan de Azúcar | *Abrothrix olivaceus* | 31 | no Ct | 0,00 | 21,98 | 0,021 | 0 |
| Pan de Azúcar | *Abrothrix olivaceus* | 32 | no Ct | 0,00 | 18,44 | 0,235 | 0 |
| Pan de Azúcar | *Abrothrix olivaceus* | 33 | no Ct | 0,00 | 17,46 | 0,457 | 0 |
| Pan de Azúcar | *Abrothrix olivaceus* | 35 | no Ct | 0,00 | 21,80 | 0,024 | 0 |
| Pan de Azúcar | *Abrothrix olivaceus* | 37 | no Ct | 0,00 | 18,71 | 0,196 | 0 |
| Pan de Azúcar | *Abrothrix olivaceus* | 38 | no Ct | 0,00 | 21,36 | 0,033 | 0 |
| Pan de Azúcar | *Abrothrix olivaceus* | 39 | no Ct | 0,00 | 17,57 | 0,425 | 0 |
| Pan de Azúcar | *Abrothrix olivaceus* | 40 | no Ct | 0,00 | 19,32 | 0,130 | 0 |
| Pan de Azúcar | *Abrothrix olivaceus* | 41 | no Ct | 0,00 | 27,95 | 0,000 | 0 |
| Pan de Azúcar | *Abrothrix olivaceus* | 42 | no Ct | 0,00 | 24,13 | 0,005 | 0 |
| Pan de Azúcar | *Abrothrix olivaceus* | 43 | no Ct | 0,00 | 19,86 | 0,090 | 0 |
| Chañaral | *Thylamys elegans* | 1 | no Ct | 0,00 | no Ct | 0,000 | 0 |
| Chañaral | *Thylamys elegans* | 2 | no Ct | 0,00 | 22,83 | 0,012 | 0 |
| Chañaral | *Thylamys elegans* | 3 | no Ct | 0,00 | no Ct | 0,000 | 0 |
| Chañaral | *Thylamys elegans* | 4 | no Ct | 0,00 | no Ct | 0,000 | 0 |
| Chañaral | *Thylamys elegans* | 5 | no Ct | 0,00 | 23,05 | 0,010 | 0 |
| Chañaral | *Thylamys elegans* | 6 | no Ct | 0,00 | 17,57 | 0,427 | 0 |
| Chañaral | *Thylamys elegans* | 7 | no Ct | 0,00 | 16,65 | 0,796 | 0 |
| Damas | *Abrothrix olivaceus* | 1 | no Ct | 0,00 | 16,94 | 0,246 | 0 |
| Damas | *Abrothrix olivaceus* | 2 | no Ct | 0,00 | 17,95 | 0,125 | 0 |
| Damas | *Abrothrix olivaceus* | 3 | no Ct | 0,00 | 17,45 | 0,175 | 0 |
| Damas | *Abrothrix olivaceus* | 4 | no Ct | 0,00 | 17,45 | 0,175 | 0 |
| Damas | *Abrothrix olivaceus* | 5 | no Ct | 0,00 | 16,67 | 0,296 | 0 |
| Damas | *Abrothrix olivaceus* | 6 | no Ct | 0,00 | 16,37 | 0,362 | 0 |
| Damas | *Abrothrix olivaceus* | 7 | no Ct | 0,00 | 18,40 | 0,092 | 0 |
| Damas | *Abrothrix olivaceus* | 8 | no Ct | 0,00 | 17,69 | 0,149 | 0 |
| Damas | *Abrothrix olivaceus* | 10 | no Ct | 0,00 | 17,59 | 0,159 | 0 |
| Damas | *Abrothrix olivaceus* | 11 | no Ct | 0,00 | 17,44 | 0,176 | 0 |
| Damas | *Abrothrix olivaceus* | 12 | 34,68 | 0,00 | 17,12 | 0,288 | <1 |
| Damas | *Abrothrix olivaceus* | 13 | no Ct | 0,00 | 18,24 | 0,102 | 0 |
| Damas | *Abrothrix olivaceus* | 14 | no Ct | 0,00 | 17,81 | 0,180 | 0 |
| Damas | *Abrothrix olivaceus* | 15 | no Ct | 0,00 | 17,54 | 0,164 | 0 |
| Damas | *Abrothrix olivaceus* | 16 | no Ct | 0,00 | 17,70 | 0,148 | 0 |
| Damas | *Abrothrix olivaceus* | 17 | no Ct | 0,00 | 17,11 | 0,219 | 0 |
| Damas | *Abrothrix olivaceus* | 18 | 32,19 | 0,03 | 17,25 | 0,200 | <1 |
| Damas | *Abrothrix olivaceus* | 20 | no Ct | 0,00 | 17,62 | 0,155 | 0 |
| Damas | *Abrothrix olivaceus* | 21 | 32,68 | 0,02 | 17,20 | 0,206 | <1 |
| Damas | *Abrothrix olivaceus* | 23 | no Ct | 0,00 | 17,70 | 0,147 | 0 |
| Santa María | *Mepraia sp.* | 1 | no Ct | NQ | ND | ND | ND |
| Santa María | *Mepraia sp.* | 2 | 45,86 | NQ | ND | ND | ND |
| Santa María | *Mepraia sp.* | 3 | 39,05 | NQ | ND | ND | ND |
| Santa María | *Mepraia sp.* | 4 | 34,38 | NQ | ND | ND | ND |
| Santa María | *Mepraia sp.* | 5 | 46,99 | NQ | ND | ND | ND |
| Santa María | *Mepraia sp.* | 6 | no Ct | NQ | ND | ND | ND |
| Santa María | *Mepraia sp.* | 7 | 37,28 | NQ | ND | ND | ND |
| Santa María | *Mepraia sp.* | 8 | no Ct | NQ | ND | ND | ND |
| Santa María | *Mepraia sp.* | 9 | 42,68 | NQ | ND | ND | ND |
| Santa María | *Mepraia sp.* | 10 | no Ct | NQ | ND | ND | ND |
| Santa María | *Mepraia sp.* | 11 | no Ct | NQ | ND | ND | ND |
| Santa María | *Mepraia sp.* | 12 | 31,84 | NQ | ND | ND | ND |
| Santa María | *Mepraia sp.* | 13 | no Ct | NQ | ND | ND | ND |
| Santa María | *Mepraia sp.* | 14 | 46,52 | NQ | ND | ND | ND |
| Santa María | *Mepraia sp.* | 15 | no Ct | NQ | ND | ND | ND |
| Santa María | *Mepraia sp.* | 16 | 46,59 | NQ | ND | ND | ND |
| Santa María | *Mepraia sp.* | 17 | no Ct | NQ | ND | ND | ND |
| Santa María | *Mepraia sp.* | 18 | 33,43 | NQ | ND | ND | ND |
| Santa María | *Mepraia sp.* | 19 | no Ct | NQ | ND | ND | ND |
| Santa María | *Mepraia sp.* | 20 | no Ct | NQ | ND | ND | ND |
| Santa María | *Mepraia sp.* | 21 | no Ct | NQ | ND | ND | ND |
| Santa María | *Mepraia sp.* | 22 | 34,56 | NQ | ND | ND | ND |
| Santa María | *Mepraia sp.* | 23 | 35,42 | NQ | ND | ND | ND |
| Santa María | *Mepraia sp.* | 24 | no Ct | NQ | ND | ND | ND |
| Santa María | *Mepraia sp.* | 25 | no Ct | NQ | ND | ND | ND |
| Santa María | *Mepraia sp.* | 26 | 44,52 | NQ | ND | ND | ND |
| Santa María | *Mepraia sp.* | 27 | 41,12 | NQ | ND | ND | ND |
| Santa María | *Mepraia sp.* | 28 | no Ct | NQ | ND | ND | ND |
| Santa María | *Mepraia sp.* | 29 | no Ct | NQ | ND | ND | ND |
| Santa María | *Mepraia sp.* | 30 | no Ct | NQ | ND | ND | ND |
| Santa María | *Mepraia sp.* | 31 | no Ct | NQ | ND | ND | ND |
| Santa María | *Mepraia sp.* | 32 | no Ct | NQ | ND | ND | ND |
| Santa María | *Mepraia sp.* | 33 | no Ct | NQ | ND | ND | ND |
| Santa María | *Mepraia sp.* | 34 | no Ct | NQ | ND | ND | ND |
| Santa María | *Mepraia sp.* | 35 | no Ct | NQ | ND | ND | ND |
| Santa María | *Mepraia sp.* | 36 | no Ct | NQ | ND | ND | ND |
| Santa María | *Mepraia sp.* | 37 | no Ct | NQ | ND | ND | ND |
| Santa María | *Mepraia sp.* | 38 | no Ct | NQ | ND | ND | ND |
| Pan de Azúcar | *Mepraia parapatrica* | 1 | 28,69 | NQ | ND | ND | ND |
| Pan de Azúcar | *Mepraia parapatrica* | 2 | 33,69 | NQ | ND | ND | ND |
| Pan de Azúcar | *Mepraia parapatrica* | 3 | no Ct | NQ | ND | ND | ND |
| Pan de Azúcar | *Mepraia parapatrica* | 4 | no Ct | NQ | ND | ND | ND |
| Pan de Azúcar | *Mepraia parapatrica* | 5 | 31,93 | NQ | ND | ND | ND |
| Pan de Azúcar | *Mepraia parapatrica* | 6 | 32,13 | NQ | ND | ND | ND |
| Pan de Azúcar | *Mepraia parapatrica* | 7 | no Ct | NQ | ND | ND | ND |
| Pan de Azúcar | *Mepraia parapatrica* | 8 | 32,23 | NQ | ND | ND | ND |
| Pan de Azúcar | *Mepraia parapatrica* | 9 | 32,49 | NQ | ND | ND | ND |
| Pan de Azúcar | *Mepraia parapatrica* | 10 | 33,89 | NQ | ND | ND | ND |
| Pan de Azúcar | *Mepraia parapatrica* | 11 | no Ct | NQ | ND | ND | ND |
| Pan de Azúcar | *Mepraia parapatrica* | 12 | no Ct | NQ | ND | ND | ND |
| Pan de Azúcar | *Mepraia parapatrica* | 13 | no Ct | NQ | ND | ND | ND |
| Pan de Azúcar | *Mepraia parapatrica* | 14 | no Ct | NQ | ND | ND | ND |
| Pan de Azúcar | *Mepraia parapatrica* | 15 | 33,43 | NQ | ND | ND | ND |
| Pan de Azúcar | *Mepraia parapatrica* | 16 | no Ct | NQ | ND | ND | ND |
| Pan de Azúcar | *Mepraia parapatrica* | 17 | 31,91 | NQ | ND | ND | ND |
| Pan de Azúcar | *Mepraia parapatrica* | 18 | no Ct | NQ | ND | ND | ND |
| Pan de Azúcar | *Mepraia parapatrica* | 19 | 31,62 | NQ | ND | ND | ND |
| Pan de Azúcar | *Mepraia parapatrica* | 20 | no Ct | NQ | ND | ND | ND |
| Pan de Azúcar | *Mepraia parapatrica* | 21 | no Ct | NQ | ND | ND | ND |
| Pan de Azúcar | *Mepraia parapatrica* | 22 | no Ct | NQ | ND | ND | ND |
| Pan de Azúcar | *Mepraia parapatrica* | 23 | 33,9 | NQ | ND | ND | ND |
| Pan de Azúcar | *Mepraia parapatrica* | 24 | no Ct | NQ | ND | ND | ND |
| Pan de Azúcar | *Mepraia parapatrica* | 25 | no Ct | NQ | ND | ND | ND |
| Pan de Azúcar | *Mepraia parapatrica* | 26 | 33,88 | NQ | ND | ND | ND |
| Pan de Azúcar | *Mepraia parapatrica* | 27 | no Ct | NQ | ND | ND | ND |
| Pan de Azúcar | *Mepraia parapatrica* | 28 | no Ct | NQ | ND | ND | ND |
| Pan de Azúcar | *Mepraia parapatrica* | 29 | no Ct | NQ | ND | ND | ND |
| Pan de Azúcar | *Mepraia parapatrica* | 30 | no Ct | NQ | ND | ND | ND |
| Pan de Azúcar | *Mepraia parapatrica* | 31 | no Ct | NQ | ND | ND | ND |
| Pan de Azúcar | *Mepraia parapatrica* | 32 | no Ct | NQ | ND | ND | ND |
| Pan de Azúcar | *Mepraia parapatrica* | 33 | no Ct | NQ | ND | ND | ND |
| Pan de Azúcar | *Mepraia parapatrica* | 34 | no Ct | NQ | ND | ND | ND |
| Pan de Azúcar | *Mepraia parapatrica* | 35 | no Ct | NQ | ND | ND | ND |
| Pan de Azúcar | *Mepraia parapatrica* | 36 | no Ct | NQ | ND | ND | ND |
| Pan de Azúcar | *Mepraia parapatrica* | 37 | no Ct | NQ | ND | ND | ND |
| Pan de Azúcar | *Mepraia parapatrica* | 38 | no Ct | NQ | ND | ND | ND |
| Pan de Azúcar | *Mepraia parapatrica* | 39 | no Ct | NQ | ND | ND | ND |
| Pan de Azúcar | *Mepraia parapatrica* | 40 | no Ct | NQ | ND | ND | ND |
| Pan de Azúcar | *Mepraia parapatrica* | 41 | no Ct | NQ | ND | ND | ND |
| Pan de Azúcar | *Mepraia parapatrica* | 42 | no Ct | NQ | ND | ND | ND |
| Pan de Azúcar | *Mepraia parapatrica* | 43 | no Ct | NQ | ND | ND | ND |
| Pan de Azúcar | *Mepraia parapatrica* | 44 | no Ct | NQ | ND | ND | ND |
| Pan de Azúcar | *Mepraia parapatrica* | 45 | no Ct | NQ | ND | ND | ND |
| Pan de Azúcar | *Mepraia parapatrica* | 46 | no Ct | NQ | ND | ND | ND |
| Pan de Azúcar | *Mepraia parapatrica* | 47 | no Ct | NQ | ND | ND | ND |
| Pan de Azúcar | *Mepraia parapatrica* | 48 | no Ct | NQ | ND | ND | ND |
| Pan de Azúcar | *Mepraia parapatrica* | 49 | no Ct | NQ | ND | ND | ND |
| Pan de Azúcar | *Mepraia parapatrica* | 50 | no Ct | NQ | ND | ND | ND |
| Pan de Azúcar | *Mepraia parapatrica* | 51 | no Ct | NQ | ND | ND | ND |
| Pan de Azúcar | *Mepraia parapatrica* | 52 | no Ct | NQ | ND | ND | ND |
| Pan de Azúcar | *Mepraia parapatrica* | 53 | no Ct | NQ | ND | ND | ND |
| Pan de Azúcar | *Mepraia parapatrica* | 54 | no Ct | NQ | ND | ND | ND |
| Pan de Azúcar | *Mepraia parapatrica* | 55 | no Ct | NQ | ND | ND | ND |
| Pan de Azúcar | *Mepraia parapatrica* | 56 | no Ct | NQ | ND | ND | ND |
| Pan de Azúcar | *Mepraia parapatrica* | 57 | no Ct | NQ | ND | ND | ND |
| Pan de Azúcar | *Mepraia parapatrica* | 58 | no Ct | NQ | ND | ND | ND |
| Pan de Azúcar | *Mepraia parapatrica* | 59 | no Ct | NQ | ND | ND | ND |

The *T. cruzi* column shows the result of satellite DNA amplification. Ct: Cycle threshold. par-eq/mL: Parasite equivalents/mL. IAC: Internal amplification control. NQ: No absolute quantification. ND: No evaluated data.
